# Metagenome Annotation Using a Distributed Grid of Undergraduate Students

**DOI:** 10.1371/journal.pbio.0060296

**Published:** 2008-11-25

**Authors:** Pascal Hingamp, Céline Brochier, Emmanuel Talla, Daniel Gautheret, Denis Thieffry, Carl Herrmann

## Abstract

The Annotathon is a novel bioinformatics teaching environment, where undergraduate students join in a community annotation effort. Besides being a rewarding educational tool, it holds the added promise of potentially useful scientific findings.

Bioinformatics is at the crossroads of different scientific disciplines, in particular biology, mathematics, and computer sciences. While this interdisciplinary aspect undoubtedly contributes to the subject's attractiveness for researchers and students, it implies the ability to master heterogeneous skills. For biology students, preconceptions lead some to believe that in silico approaches are for computer-savvy specialists only. Teaching bioinformatics not only implies helping students to overcome perceived obstacles, such as mastering biostatistics or computational tools. It also requires momentous efforts to effectively drive home the message that applying bioinformatics tools and interpreting their results is an eminently biological endeavor.

With bioinformatics progressively entering core life sciences curricula, these challenges are being faced by an increasing number of universities. Luckily, since most bioinformatics resources are accessible online, almost every type of bioinformatics teaching can be done from a computer room equipped with broadband internet. Early on in their undergraduate studies, students can tackle bioinformatics questions that are at the forefront of current research, and even investigate problems that have not been addressed to date. Another advantage of in silico teaching is that processes are relatively fast and can be repeated over and over again at little cost, which encourages the learning process through “trial and error” iterations. However, easy data generation has its pitfalls: raw data flooding and, more perniciously, overconfidence in predictions. The current feeling that “turning data into knowledge” [1] is the major bottleneck in science in general, and particularly in postgenomic bioinformatics, is of immediate and extreme pedagogical importance.

In this report, we present our experience using public cutting-edge genomic data, combined with a newly developed online environment for teaching bioinformatics at undergraduate level. The approach combines the excitement of novelty provided by “hot-off-the-sequencer,” as yet non-annotated metagenomics data, with a highly structured e-learning Web tool. We discuss its practical use in class together with assessments made by students.

## Course Overview

The teaching approach we present is the result of several years of experience in teaching bioinformatics at undergraduate level, during which we identified the following points as key ingredients for a successful teaching approach:

Learning by doing: at undergraduate level, bioinformatics is best introduced by first-hand experience; theoretical considerations are easier to grasp once students are truly familiar with the tools.

Learning through repetition: mastering bioinformatics tools requires using and reusing them in a wide range of situations. Confronted with “twilight zone” similarities, or “garbage in, garbage out” phylogenetic tree reconstructions, students experience both the potential and the real limits of in silico approaches.

Learning through excitement: a powerful incentive for students is the projection at the very frontiers of knowledge, even if this can lead to uncomfortable situations, where analysis results—a priori unknown even to instructors—can prove difficult to interpret.

Learning from constructive criticism: giving students the opportunity to correct themselves results in accelerated progression over time.

### Tasks assigned to the students.

The goal of the course is to teach students how to computationally annotate biological sequences (DNA and protein sequences). The starting point is a short stretch of DNA sequence (such as a single metagenomic sequencing read) that students are asked to study according to two major lines of inquiry: (1) prediction of gene product putative function and (2) prediction of taxonomic group of origin.

**Figure 1:**
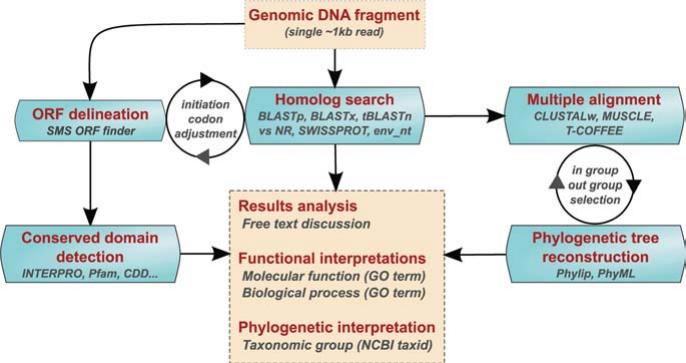
Annotation Work Flow Starting with a short DNA metagenomic sequencing read, students predict both functional and phylogenetic classifications using classical bioinformatics analyses.

The in silico analyses (Figure 1) classically begin with open reading frame (ORF) prediction, followed by identification of conserved protein functional domains, as well as similarity searching in sequence databases. Where homologs are identified, analysis concludes with multiple sequence alignments and phylogenetic tree reconstruction.

Students are expected to apply this workflow to several distinct sequences (typically three, randomly sampled), and are therefore likely to encounter a range of different cases. Since even for this limited number of sequences, the annotation effort requires stamina, we have called this procedure “Annotathon.”

For each step of the analysis, one or several standard Web tools are suggested, and when different algorithms are used, the discussion of any observed discrepancies is part of the task. Raw results (e.g., Basic Local Alignment Search Tool [BLAST] reports, etc.) as well as student interpretations (e.g., gene ontology [GO] term assignments) are collected through the Annotathon dedicated Web interface, which also manages all aspects of course progression (see “Annotathon Online Work Environment”). Analytic and argumentative skills are invoked in a final synthesis, where students are required to rigorously describe how each result supports proposed hypotheses.

### Interaction with instructors.

**Figure 2:**
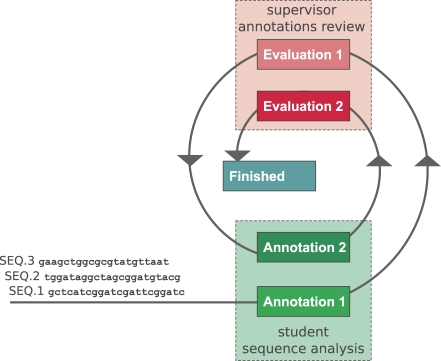
Two-Stage Sequence Annotation Cycle Initial student annotations are reviewed the first time by instructors. Students take advantage of this review to improve their analyses and produce a final annotation, which is reviewed a second time; each review is accompanied by a quantitative evaluation. This process is repeated for *n* sequences (here *n* = 3).

After initial tutorials led by instructors in the computer room, students complete their assignments autonomously and at their own pace. Interaction and feedback from the instructors outside class is carried out almost in real time, using dedicated online forums and chats. More importantly, students benefit from a progressive evaluation cycle (Figure 2) that allows them to respond to instructor constructive criticism by editing and improving their first-pass annotations. While this procedure clearly increases the burden on instructors, we have found that it accelerates student progression.

### Data sources.

We have chosen Global Ocean Sampling (GOS, [2–4]) metagenomic sequences as our data source. While this is an almost inexhaustible source of data, it is not integrated in the default sequence databases of most online tools, such as the National Center for Biotechnology Information (NCBI)'s “NR” non-redundant compilation. Furthermore, when available, the environmental sequence databases (such as “ENV” at NCBI) do not usually provide any annotation other than submitter identity and sampling location. The novelty and biodiversity aspect of these projects undoubtedly contributes to positive student perception [5], but any other source of sequences with no public annotations can be exploited. Indeed, our first 2005 Annotathon campaign used contigs from an ongoing Kluyveromyces thermotolerans genome sequencing project. The tentative taxonomic classification of the source organism, which is the aim of the phylogenetic analysis in the case of metagenomic sequences, shifts to identification of ortho- and paralogs in the case of genome sequences of known origin. Since the annotation process is focused on protein coding genes, we prefiltered the GOS dataset to exclude sequences that do not contain at least a 60 amino acid ORF, and broke up the yeast contigs 50–100 bp upstream and downstream of ORFs over 60 amino acids long.

## Annotathon Online Work Environment

To ensure students become familiar with the tools of the trade, all sequence analyses per se are carried out using the traditional online tools, made available by bioinformatics resource providers such as NCBI, the European Bioinformatics Institute, or Phylogeny.fr. The overall work progression, however, is controlled through the Annotathon dedicated Web environment, which manages every step from sequence distribution and results collection through to instructor evaluations. Together with its detailed annotation guidelines and team communication tools, the Annotathon provides students with a structured work environment that buffers the plethoric and heterogeneous network of bioinformatics tools that often daunts the uninitiated.

**Figure 3:**
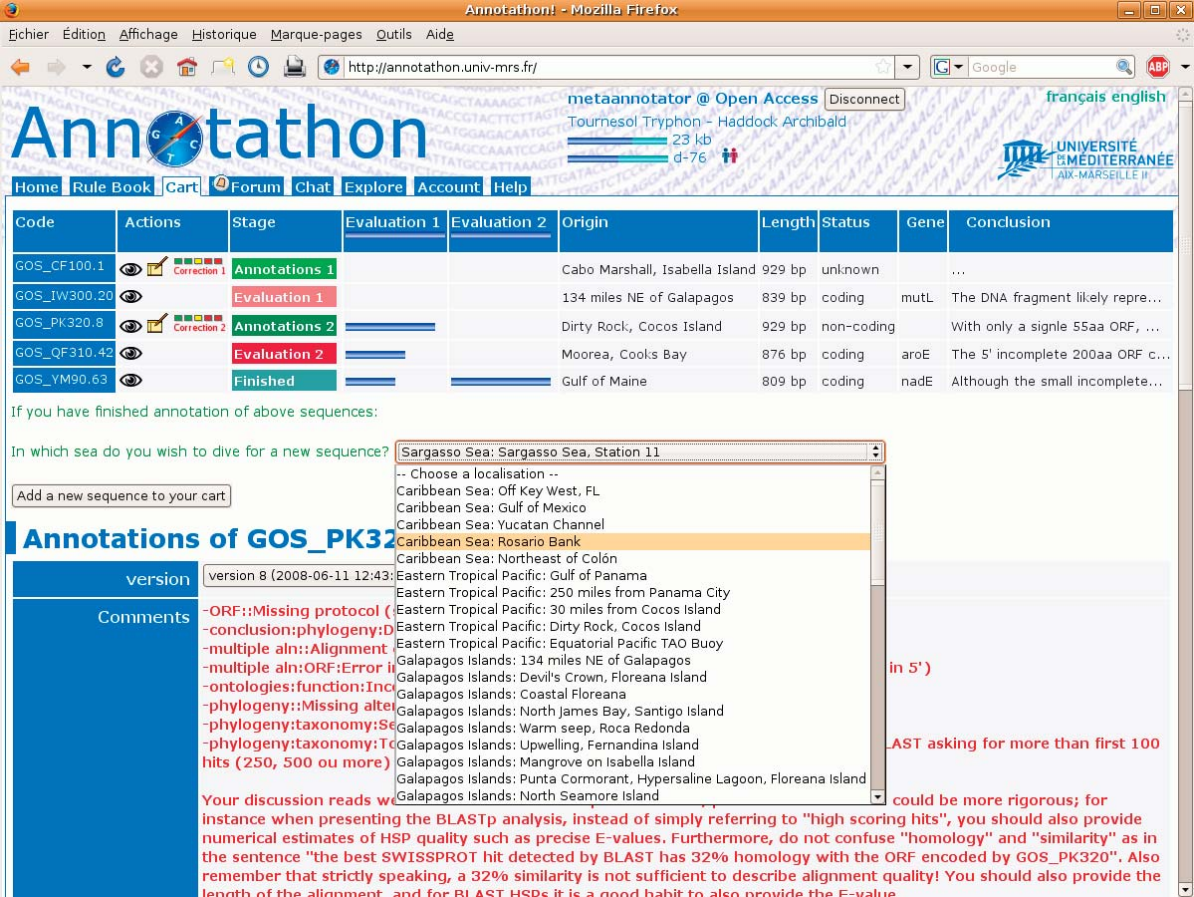
Annotathon Sequence Cart The five DNA fragments, assigned to a student, illustrate each possible annotation stage: ongoing initial “Annotations 1,” awaiting initial “Evaluation 1,” ongoing final “Annotations 2,” awaiting final “Evaluation 2,” and sequence annotations “Finished.” Quantitative evaluations are represented as blue horizontal bars, and an example of qualitative evaluations is shown in red in the “Comments” field associated with each sequence record. The popup menu in the center shows GOS sampling locations, from which sequences can be added to the cart.

The Annotathon environment is centered around a sequence cart, which students progressively load with new raw sequences picked from various ocean locations (Figure 3). Raw analysis results (e.g., BLAST reports) are stored in specific Annotathon fields, followed by student interpretations (e.g., ORF location and GO terms). The Annotathon instructor control panel provides evaluation management tools to help efficient assignment of both qualitative comments and quantitative marks (Figure S1). At the end of the course, marks for each annotation stage are weighted to help cancel out discrepancies in sequences analysis complexities, normalized across instructors, and compiled into an overall grade for each participating student. Quality annotations can be directly exported to the dedicated “Metagenes” wiki companion Web site, providing public interactive access to annotated sequences (http://biologie.univ-mrs.fr/Metagenes/).

Team communication outside supervised classes relies on responsive online forums where students are also encouraged to reply to fellow student questions. The team home page displays progression meters (e.g., kilobases collectively annotated), evaluation-based instantaneous team rankings, and scientific summaries, including taxonomic and molecular function distributions (Figure S2).

Teachers wishing to use the Annotathon for their courses are invited to create new teams on the public server at http://annotathon.univ-mrs.fr/ (course logistics and team management are detailed in the instructor manual:http://annotathon.univ-mrs.fr/Metagenes/index.php/Instructor_Manual). The underlying open-source software (PHP and MySQL scripts, under a General Public License) is also available for local installation (https://launchpad.net/annotathon/). In addition, a special “Open Access” team is available for freelance students (volunteer instructors are most welcome to help oversee the Open Access team).

## Practical Implementation in Class

### Format of the course.

We have applied this teaching method to bioinformatics courses, amounting to three of the 30 credits that make up a semester, mainly at the Université de la Méditerranée and Université de Provence (Marseille, France). The target audience consists of third-year Bachelor of Science students (B.S., French “*Licence*”) majoring in cellular biology or biochemistry, most of whom have a basic background in bioinformatics through a three-credit introduction to bioinformatics unit in their second year. The obligatory courses include ten hours of theoretical teaching, followed over a five-week period by four half-day practical sessions in the computer room. It is made clear to students that annotations need to be continued, at will, outside classes; connection logs indeed show that students spend on average 42 hours online, of which only 16 correspond to supervised classes. For practical work, all students work in pairs, sharing a unique Annotathon account. The final course grade awarded to each student is split equally between the practical evaluations, directly provided by the Annotathon, and an individual theoretical exam.

### Main difficulties encountered.

Students appear to be very receptive to the format of the course and its practical dimension. Most apprentice annotators are excited by the perspective of contributing, however modestly, to the creation of knowledge.

A first hurdle is the delineation of ORFs. Students have difficulties understanding the logic for the choice of the initiation codon. We ask them to use a loose condition (“any initiation codon”), as short read coding sequences might have been truncated in their 5′ region, but insist on the fact that they should come back later to adjust the ORF start position as necessary, especially after multiple alignment with homologs; failure to do so is a recurrent critique by instructors and is mentioned by students as one of the difficulties they have encountered.

Further hurdles are usually connected with multiple alignment and phylogenetic analysis. Many students are destabilized by the “trial and error” nature of phylogenetic analyses. The sequence selection strategy for multiple alignment is difficult to grasp for most. The main mistakes are (1) selecting overly similar sequences, (2) including nonhomologous sequences in the multiple alignment, (3) improperly defining in- and out-groups, or (4) failing to properly sample the taxonomic landscape. The two first mistakes are usually the result of extreme BLAST cases, either highly evolutionarily conserved protein sequences or ORFans. Furthermore, in the absence of handy quality scores or E-values, students often find it difficult to judge the quality of a multiple alignment and regularly fail to identify sequences that should be removed from a suboptimal alignment. Multiple alignment interpretation is often superficial, and few students confront the conserved regions, identified in multiple alignments, with identified protein domains or known family structural features.

The construction and interpretation of phylogenetic trees is the single most challenging in silico analysis faced by students. They commonly stumble over whether the trees obtained are compatible with known reference phylogeny (co-clustering of same taxon sequences), or if trees obtained by alternative methods are congruent. Evolutionary events like duplications or horizontal gene transfers are frequently missed.

Unexpectedly, we have found that many students have considerable difficulties summarizing their findings in their final conclusion and producing a rigorous argumentation. This is supported by poll results (see “Feedback from students” below), which show that students consider the writing of the conclusion the most arduous part of the assignment. This was a very discriminative point among students, some showing truly remarkable skills, while others remained at a very basic level in their analysis.

### Scientific findings.

**Figure 4:**
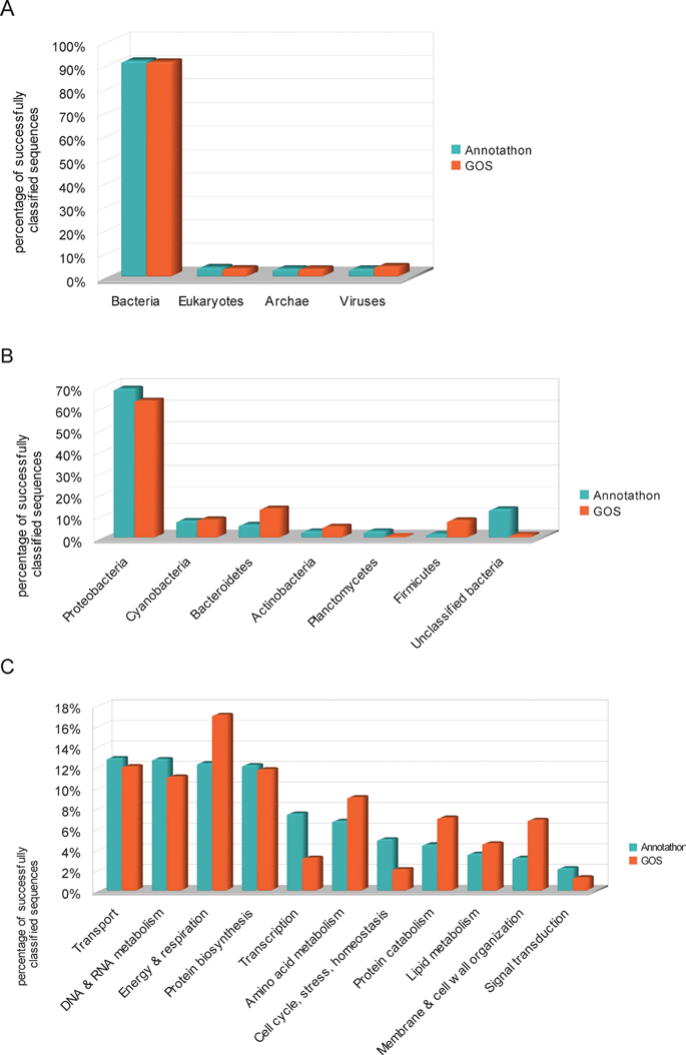
Comparison of Student (Annotathon) Versus Literature (GOS) Annotations (A) Taxonomic domain-level classifications, determined either by students using phylogenetic tree reconstructions (Annotathon, 182 sequences classified) or by an automatic BLAST-based scheme (GOS, 5,058,757 sequences classified [4]). (B) Distribution of sequences across bacteria taxa. Proportions are determined either by students using phylogenetic tree reconstructions (Annotathon, 182 sequences classified) or by 16S-based estimates (GOS, 4,125 sequences classified [3]). (C) Gene products functional classifications. Proportions are determined either by students (Annotathon, 685 sequences classified) or by automatic TIGR (The Institute for Genomic Research) role assignment (GOS, 760,659 sequences classified [2]). Only categories from each partition that could be matched are shown.

The 515 students that have taken part in the Annotathon over the past three years have analyzed a total of 2.3 Mb of ocean microbial DNA, representing 9,500 hours of cumulative annotation. Considering the relatively modest number of sequences hand-annotated so far and the limited experience of apprentice annotators, the overall Annotathon scientific conclusions are surprisingly close to the large-scale automatic analyses published in the literature [2–4]. Indeed, domain-level taxonomic classifications are essentially indistinguishable, with bacteria representing over 90% of the sampled DNA (Figure 4A), and proteobacteria overabundance at around 65% (Figure 4B). Over 60% of sequence gene products were assigned functional categories by students, but comparison of Annotathon versus GOS functional assignments is more difficult, since two distinct ontologies have been used (Figure 4C). Even so, there is unexpectedly high concordance concerning the four most abundant biological processes that keep ocean microbes busy: transport, energy, DNA metabolism, and protein synthesis, each representing 10% to 20% of assigned known functions.

### Feedback from students.

We carried out a survey among students who participated in the Annotathon 2007–2008 campaigns, and collected 60 responses from a total of 117 participating students (Text S1). Since students were free to participate or not in the survey, we cannot exclude a bias toward highly motivated students.

We first asked students to evaluate their skills in sequence analysis and annotation, before and after the course. The amplitude of progress felt by students during the course exceeded our expectations. Although self-assessed student experts might suffer from a little overconfidence, this shows a welcome increased self-assurance in a subject traditionally considered difficult by biologists.

Interestingly, when asked which part of the assignment was the most difficult, a majority ranked the writing of the conclusion as the most difficult part of the whole exercise, while phylogenetic analysis ranked second. This was consistent with our impression from reviewing annotations, but we did not anticipate that students would share our point of view.

During the supervised practical sessions, several tutors were present in the computer room to guide students. Faced with the results of an analysis, different instructors would frequently offer slightly divergent interpretations of the results. We wanted to know how students reacted to this situation, often their first contact with research science as opposed to textbook science: a majority found the situation “surprising,” but few (6%) declared that this was shocking. It is pleasing to note that 20% of the students found this to be “instructive.” Indeed, we believe that it is of great importance for future scientists to experience and acknowledge the fact that most of the time, researchers are faced with multiple possible explanations for a single observation.

Since our approach relies heavily on the concept of “learning through repetition,” we asked students whether they found this aspect to be important in the learning process. An overwhelming number of students indeed found this to be the case, with 94% of the students finding repetition to be useful, very useful, or indispensable.

Overall, the survey shows that students responded very positively to this new approach to bioinformatics teaching, including the opportunity to annotate anonymous metagenomic sequences (71% of the students found this aspect “stimulating”). An encouraging survey message is that while 75% of the students found the exercise difficult, the same percentage of students declared that they retained a positive impression of bioinformatics as a consequence of following the course.

## Conclusions and Perspectives

So far, we have mainly focused on the educational aspects of our approach, but the encouraging correlation between student hand-crafted and large-scale automatic annotations shows potential for pushing a step further. Could we envisage that student annotations be made public, contributing to a long-term international distributed annotation jamboree of large (meta)genomics datasets? This exciting possibility would undoubtedly be welcomed as a further incentive by participating students [6], and could even yield useful, if modest, scientific contributions. Final annotation quality control by instructors could be simplified by having several independent groups of students redundantly annotate the same sequences and by filtering for converging GO and taxonomy annotations before public release. Similar distributed annotation efforts have been applied to literature curation for DNA-binding data [7], and were just recently implemented in the Gene Wiki [8], WikiPathways [9], and WikiProteins [10] systems to encourage community annotation of genes, pathways, and proteins, respectively.

Having run this course since 2005, our impression—corroborated by student feedback—is that this teaching approach is far more successful than our previous methods, based on canned re-annotation of a few classic, predictable, well-known sequences. By the end of the course, we were very impressed by the familiarity of students with the core in silico tool box, running BLASTs at the drop of a hat and discussing E-values naturally. Joining many others [11–14], we recognize the benefit of exposing students to real research situations early on in their training. Left until later, the no less crucial opening of the algorithmic black box [15] will be facilitated by a positive and confident student mindset.

## Supporting Information

Figure S1 Instructor Evaluation Tools (A) Predefined comments: in the case of common student mistakes, instructors can simply tick appropriate boxes. (B) Free text fields are available for more specific criticisms. (C) To help assess how students have responded to the initial review, instructors can view a comparison between student initial and final annotations (deletions are crossed out in blue, insertions are highlighted in red).
(7.7 MB EPS).

Figure S2 Student Functional and Taxonomic Classifications of Metagenome DNA Fragments For each GO biological process, GO molecular function, and taxonomic classifications, the left column diagram shows the proportion of metagenome fragments that could be assigned to a known category, while the right diagram represents the detailed distribution of successfully classified sequences. Data compiled from the Marseilles Cellular Biology and Biochemistry teams during the 2007 and 2008 Annotathon campaigns (strict NCBI-based taxonomy classification was only introduced in 2008, which explains the lower total number of taxonomy classifications).
(25 KB PNG).

Text S1 Student Exit Survey This document presents the survey protocol, survey text, and complete numerical results, as well as selected graphical representations of responses.
(136 KB PDF).

Text S2 List of Students who Contributed to Annotathon Development by Taking Part in the 2005–2008 Maiden Campaigns (67 KB PDF).

## Abbreviations

BLAST: Basic Local Alignment Search Tool
GO: gene ontology
GOS: Global Ocean Sampling
NCBI: National Center for Biotechnology Information
ORF: open reading frame
