# Supplementary figures and images for: Metagenome Annotation Using a Distributed Grid of Undergraduate Students
Source: PLoS Biol. 2008 Nov 25;6(11):e296. doi: 10.1371/journal.pbio.0060296 (PMC2586363; doi:10.1371/journal.pbio.0060296)

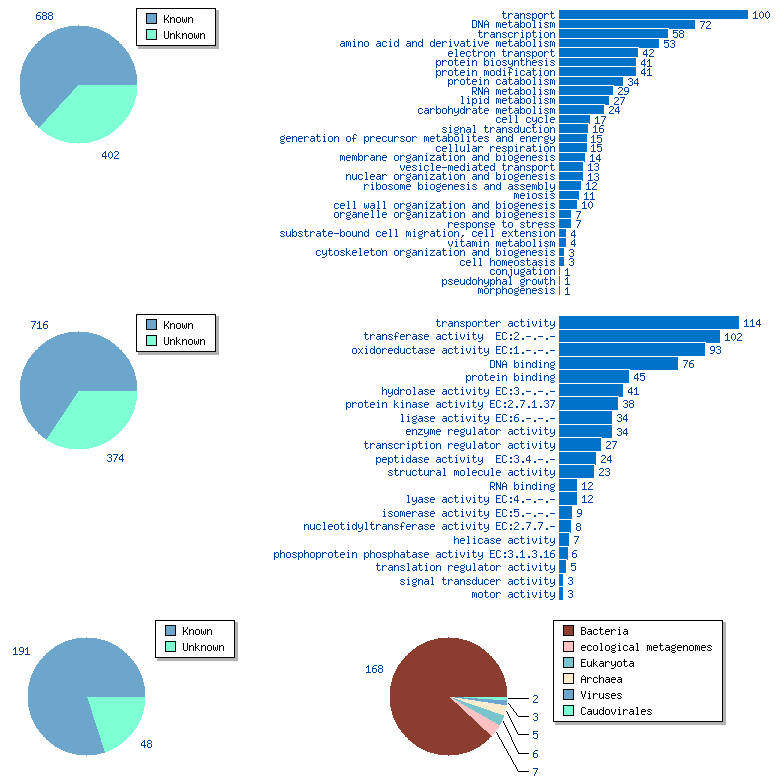

Supplement: Figure S2 — For each GO biological process, GO molecular function, and taxonomic classifications, the left column diagram shows the proportion of metagenome fragments that could be assigned to a known category, while the right diagram represents the detailed distribution of successfully classified sequences. Data compiled from the Marseilles Cellular Biology and Biochemistry teams during the 2007 and 2008 Annotathon campaigns (strict NCBI-based taxonomy classification was only introduced in 2008, which explains the lower total number of taxonomy classifications). (25 KB PNG). [file pbio.0060296.sg002.png]
