# Supplementary material for: Metagenome Annotation Using a Distributed Grid of Undergraduate Students
Source: PLoS Biol. 2008 Nov 25;6(11):e296. doi: 10.1371/journal.pbio.0060296 (PMC2586363; doi:10.1371/journal.pbio.0060296)
Supplement: Text S1 — This document presents the survey protocol, survey text, and complete numerical results, as well as selected graphical representations of responses. (136 KB PDF). [file pbio.0060296.sd001.pdf]

Supplementary Text S1

Student Exit Survey

We decided to conduct a student survey in order to evaluate how students reacted to this new pedagogical experience. The purpose was to *(i)* get a feedback in order to improve some aspects of the Annotathon environment, and *(ii)* validate the concept and its effectiveness.

Protocol

The pool of students surveyed consisted of 117 students who participated in the Annotathon in the fall 2007 and spring 2008 courses, some of which have a major in biochemistry while others have a major in cellular biology. The Annotathon is run as part of an obligatory course for all undergraduate biology students.

The survey consists of 31 questions, plus some free text fields for comments. The language was French, and we used the free software package Limesurvey (http://www.limesurvey.org/) installed on our server to perform the survey online. A personal email invitation was send to each of the 117 students, followed by 2 reminders at 1 week interval for those who had not replied so far. Students took the survey after being informed of their grades for the teaching unit. We thus collected 60 complete anonymous responses (51%). Some students had completed the Annotathon several month ago, which might have affected the response rate.

	**Question**	**Possible answers: number of answers**
1	What is your biology major?	• cellular biology: 35<br>• biochemistry: 20<br>• other: 5
2	Would you say that making the Annotathon accessible outside the tutored practical session was:	• a good idea: 59<br>• a bad idea: 1
3	Was internet access outside class a hurdle?	• never: 33<br>• rarely: 10<br>• from time to time: 12<br>• often: 2<br>• permanently: 2<br>• no opinion: 1
4	Do you have a private internet access?	• yes: 54<br>• no: 6
5	Did you use the free internet service on the campus during the Annotathon ?	• never: 11<br>• rarely: 15<br>• from time to time: 22

		• often: 9<br>• permanently: 2<br>• no opinion: 1
6	How many hours did you spend outside tutored sessions for the Annotathon ?	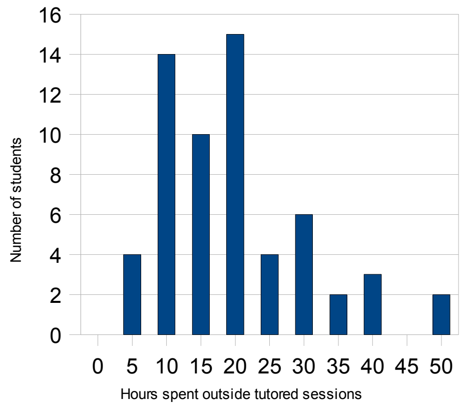
7	How would you rate your general abilities in using computers (internet browsing, file handling,...) **before and after** the Annotathon ?	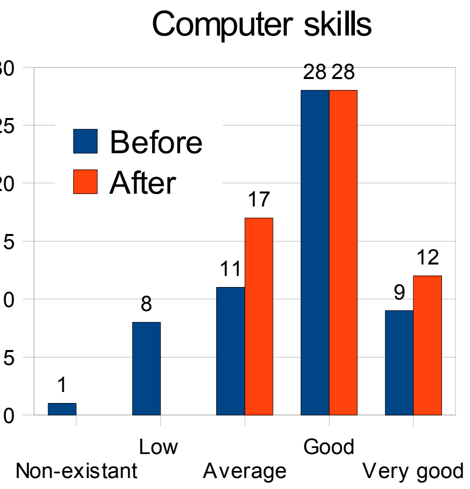
8	On a 0 (non-existant)-5 (expert) scale, how would you rate your expertise in sequence annotation **before and after** the Annotathon sessions ?	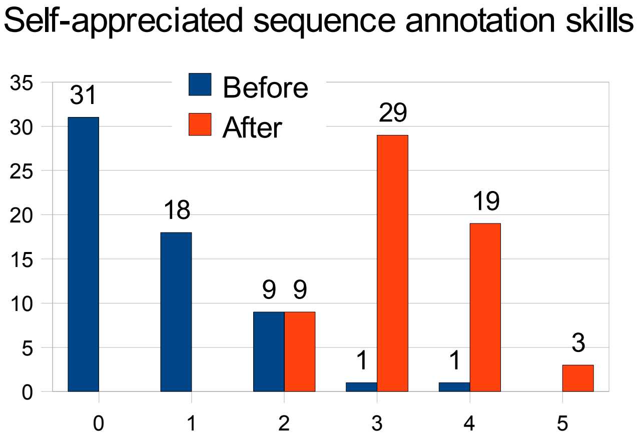
9	Which bioinformatics tools had you	• ORF finder: 29

	already used prior to the Annotathon ? *(multiple answers allowed)*	• Blastp: 37<br>• tblastn: 17<br>• blastx: 22<br>• clustalw: 10<br>• phylip: 3<br>• InterPro: 16
10	Rank the following list of tasks performed during the Annotathon, from the easiest (1) to the most difficult (7): *(ranking is based on a majority rule, numbers indicate how many students assigned the corresponding rank)*	1. ORF finding: 45<br>2. start codon identification: 24<br>3. finding homologs with Blast: 19<br>4. identification of functional domains: 24<br>5. analysis of MSA: 25<br>6. phylogenetic analysis: 26<br>7. writing the conclusion: 30
11	Which of these task do you still find the most challenging after completion of the Annotathon? *(multiple choices allowed)*	• ORF finding: 2<br>• start codon identification: 6<br>• finding homologs with Blast: 6<br>• identification of functional domains: 3<br>• analysis of MSA: 21<br>• phylogenetic analysis: 40<br>• writing the conclusion: 21
12	Would you say that getting started with the Annotathon interface was:	• easy: 13<br>• average: 33<br>• difficult: 12<br>• very difficult: 1<br>• N/A: 1
13	Do you find the Annotathon interface easy to use ?	• absolutely not: 2<br>• little: 5<br>• average: 42<br>• very: 11
14	Please rate the usefulness of the following tools: discussion forum, FAQ section, rulebook, summary of annotation statistics:	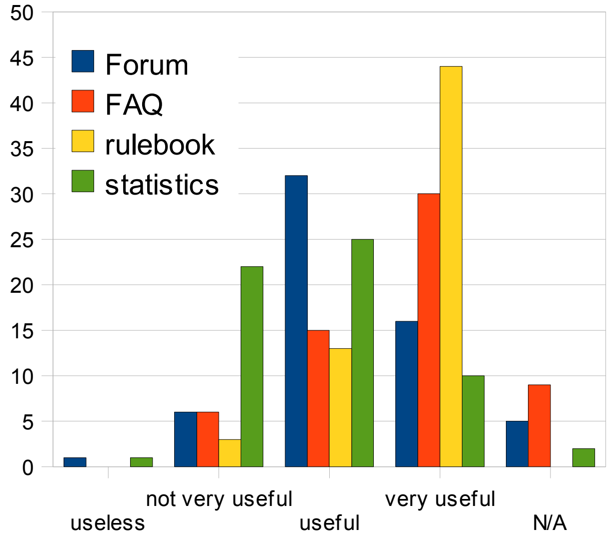

15	Please rate the usefulness of the following possible future improvements:<br>• simultaneous connection on the same account<br>• online chat	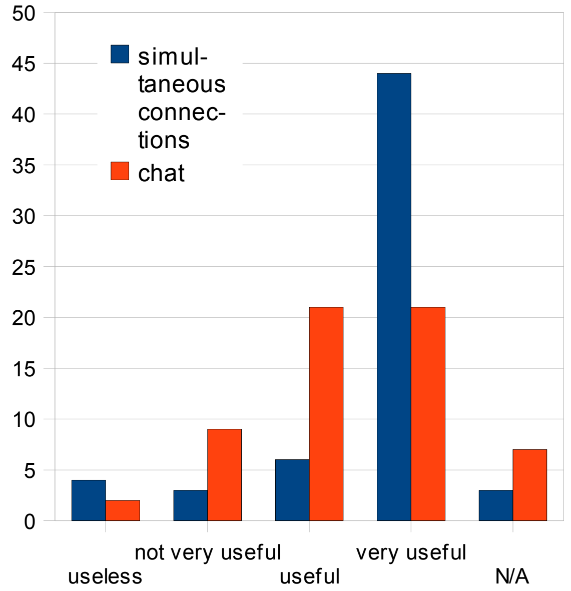
16	Didi you find it useful to be able to collect the opinion of distinct tutors during the tutored sessions ?	• yes: 44<br>• no: 9<br>• N/A: 7
17	How do you judge the fact that some tutors might have divergent opinions in certain cases ?	• choking: 4<br>• surprising: 25<br>• normal: 16<br>• instructive: 11<br>• N/A: 4
18	You do you find the “double rating” system :	• useless: 2<br>• little useful: 1<br>• useful: 13<br>• very useful: 24<br>• indispensable: 16<br>• N/A: 4
19	Did the criticism from the evaluators help you to improve your skill ?	• absolutely not: 1<br>• little: 5<br>• average: 36<br>• very much: 18
20	Do you find the amount of work:	• excessive: 32<br>• reasonable: 24<br>• adapted: 4

		• insufficient: 0
21	You do you react to the fact that you were working with so far unannotated sequences?	• stimulating: 44<br>• no particular reaction: 12<br>• confusing: 1<br>• choking: 0<br>• N/A: 3
22	Do you find it interesting that your annotations might be made publicly available:	• no interest: 3<br>• little interest: 1<br>• interesting: 23<br>• very interesting: 12<br>• I did not know about this possibility: 19<br>• N/A: 2
23	As a conclusion, did you find these practical sessions:	• passionating: 7<br>• interesting: 45<br>• not interesting: 3<br>• N/A: 5
24	As a conclusion, did you find these practical sessions:	• very easy: 0<br>• easy: 8<br>• difficult: 42<br>• very difficult: 5<br>• N/A: 5
25	As a conclusion, did you find these practical sessions:	• very useful: 6<br>• useful: 42<br>• little useful: 5<br>• useless: 0<br>• N/A: 7
26	Do you find that annotating 3 sequences is:	• insufficient: 1<br>• sufficient: 47<br>• excessive: 11<br>• N/A: 1
27	Do you think that repeating the same steps several times is:	• useless: 0<br>• little useful: 2<br>• useful: 28<br>• very useful: 20<br>• indispensable: 6<br>• N/A: 4
28	Do you think that the skills you acquired during these sessions will be useful for your future:	• yes: 37<br>• no: 14<br>• N/A: 9
29	Did these Annotathon session give you a positive impression of bioinformatics:	• yes: 41<br>• no: 5<br>• neutral: 14

30	Did these Annotathon session encourage you to study bioinformatics further:	● yes: 10<br>● no: 36<br>● neutral: 14
31	Did these Annotathon session encourage you to start a master in bioinformatics:	● yes: 12<br>● no: 42<br>● neutral: 6

Free comments (French with tentative translation).

- Il faudrait une séance de td en plus ou une séquence en moins avec la possibilité de faire la troisième en tant que bonus
  There sould be an additional practical session or a sequence less to annotate with the possibility of annotating the third as a bonus

- Le travail hors TD a été énorme. Il est parfois dur aussi de trouver le temps car la bioinformatique n'est qu'une matière parmi de nombreuses autres dans le cursus.
  The work outside TD has been enormous. It is also sometimes hard to find time because bioinformatics is a course among many others in the curriculum.

- Heureusement que l'Annotathon était accessible en dehors de heures de td, sinon il aurait été beaucoup trop long et impossible à finir pour le seul temps des heures de td.
  Fortunately the Annotathon was accessible outside tutored session, otherwise it would have been much too long and impossible to finish within the tutored sessions alone.

- Intéressant mais prend énormément de temps pour être bien fait surtout pour la moitié de la note d'une matière à trois crédits.
  Interesting but takes considerable time to be done well, especially when considering that it counts 50% of a 3 credit course.

- Le but de l'Annotathon n'est pas très clair et je n'arrive pas à voir dans quel contexte l'utiliser exactement en biochimie.
  The purpose of the Annotathon is not very clear and I can not see in what context to use it exactly in biochemistry.

- C'est cool !

- Je déplore le problème des binômes qui ne travaillent pas autant l'un que l'autre
  I regret the problem of pairs of students where both do not work as much as one another

- La partie phylogénie est très difficile, beaucoup de travail a la maison au détriment des autres matières. Un excellent site
  The phylogeny is very difficult, lot of work at home at the expense of other subjects. An excellent site

- Rythme soutenu selon les séquences piochées
  Highly demanding depending on the sequences drawn.

- Meilleure matière de l'année et je suis sincère ...
  Best course of the year and I mean it...

- Je trouve que 30 heures en dehors des TD c'est un peu trop!
  I think 30 hours outside the TD is a bit too much!

- Manque d'encadrement pendant les séances de TD malgré le nombre d'étudiants assez importants. Certains encadrants n'étaient pas assez clairs ou manquaient de connaissances ou oubliaient des précisions importantes.
  Insufficient supervision during the practicals in view of the important number of students. Some tutors were not clear enough or lacked knowledge or forgot important details.

- Pour moi l'analyse phylogénétique reste l'étape la plus difficile, en particulier le choix d'un groupe extérieur.
  For me the phylogenetic analysis remains the most difficult step, especially the choice of a external group.

- La quantité de travail personnel à fournir nous permet de bien nous habituer à tous les outils de bioinfo et en général à la fin on est "calé sur le sujet"!
  The amount of work required has allowed us to get used to all the bioinformatic tools and usually at the end we are "familiar with the topic"!

- Je pense que les cours sur la phylogénie n'étaient pas vraiment appropriés aux TD. En effet, les cours ont été très théoriques, trop en longueur sur des points qui, une fois devant notre Annotathon, ne nous ont servi à rien. Savoir comment un logiciel faisait un arbre ne m'a absolument pas aidé pour savoir comment construire mon arbre, comment l'interpréter ou savoir quelles modifications faire pour avoir un arbre plus judicieux !! Je pense que des cours d'analyse (exemples de résultats, interprétations, remarques, info à tirer ...) m'aurait beaucoup aider pour faire mes analyses !
  I think that courses on the phylogeny were not really appropriate for the practicals. Indeed, teaching has been very theoretical, too lengthy on points which, once in front of our Annotathon, were of little use. Knowing how software builds a tree did absolutely not help to learn how to build my tree, how to interpret it or what changes to make to get a better tree! I think that learning how to analyze results (examples of results, interpretations, remarks, information to retrieve ...) would have been much more helpful for my analysis!

- Sur la conclusion et les diverses analyses, comme il y avait des divergences dans les exigences des correcteurs, il était difficile de savoir exactement ce qu'il fallait ou ne fallait pas mettre.
  On the conclusion and the various analyses, as there were differences in the requirements made by the evaluators, it was difficult to know exactly what to include or not.

- J'ai déjà dit que j'avais bien aimé la matière ?
  Did I already mention that I liked this course?

- Le travail en groupe est souvent un problème car difficulté de trouver des créneaux de travail en commun: la possibilité de se connecter en même temps faciliterait certains problèmes.
Working in pairs is often a problem because of difficulty finding slots to work in common: the ability to connect at the same time would facilitate some problems.

- La rubrique statistique avec l'affichage des notes entraîne une "concurrence" entre les binômes, est-ce bien nécessaire ?? Le forum n'est pas pratique, nous devrions pouvoir poser directement les questions dessus, le chat m'a été inutile ... La connections simultanée sur un même compte peut être discriminatoire pour les binômes qui ne possèdent qu'une connexion.
The statistics with the display of grades leads to a "competition" between pairs, is this really necessary? The forum is not easy to use, we should be able to ask questions directly, the chat was not useful for me ... The simultaneous connections on the same account may be discriminatory for pairs who do have an internet connection.

- Ca devrait être le même prof qui corrige la correction 1 et 2 d'un même séquence car les remarques sont personnelles à chaque prof et parfois divergente entre les prof.
It should be the same teacher who performs the correction 1 and 2 of the same sequence as the remarks are personal to each teacher and sometimes divergent between evaluators.[Instructor comment: corrections 1 and 2 are indeed always performed by the same instructor]

- Une rubrique statistique plus détaillée serait la bienvenue ainsi qu'une meilleurs cohérence entre les profs concernant le barème pris en compte pour la notation des conclusions.
More detailed statistics would be welcome and a better consistency between teachers on the scale taken into account in the rating of conclusions.

- Plus de détails sur l'étude phylogénétique
More details about the phylogenetic analysis

- Il faudrait définir un cahier des charges en plus des règles du jeu. Ainsi tout le monde saurait exactement ce qui est demandé et nécessaire.
One should precisely define specifications in addition to the rulebook; this way everyone would know exactly what is required and necessary.

- La quantité de travail hors des TD est énorme lorsque l'on a aucune connaissance en Annotathon, peut être faire deux séquences au lieu de trois serait bénéfique .
The amount of work outside the TD is enormous when one has no knowledge of Annotathon, maybe annotating two sequences instead of three would be beneficial

- Avoir plus d'encadrants lors des TD. Et des explications communes seraient bien parce que si chaque encadrant dit quelque chose de différents...
Have more tutors during practical sessions. And common explanations because if each supervisor says something different ...

- J'ai vraiment apprécié les TD Annotathon parce ce sont des cours qui changent des cours magistraux. Mais il pourrait être utile de rajouter un séance supplémentaire de TD et de mieux informer les étudiants sur les possibilités d'accéder aux salles informatiques en dehors des

heures de cours.
I really appreciated the Annotathon practicals because they are a welcome alternative to lectures. But it might be useful to add an additional practical session and better inform students about the possibilities to access computer rooms outside teaching hours.

- Le seul problème que j'ai eu, c'est que lors des correction il m'est arrivé que le correcteur et l'enseignant présent lors des séances de TD se sont contre dit et cela s'est ressentit dans la notation.
The only problem I had was that during the correction it happened that the correction and the teacher present at the tutored sessions contradict each other and it was felt in final marking.

- Je voulais avant faire bioinfo, mais apres avoir travaillé avec ces professeur, ils m'ont coupé l'envie malheureusement.
I wanted to do before bioinfo, but after having worked with the teachers, they put me off unfortunately.

- Nous découvrons beaucoup de choses au cours des TP sur le fonctionnement des annotations, les enseignants devraient plus prendre en compte le fait que pour la plupart c'est notre première expérience dans le domaine et que nous sommes relativement perdus ...
We discover many things during the practicals on the functioning of annotations, teachers should take more into account the fact that for most this is our first experience in the field and we are relatively lost ...

- Il est intéressant d'avoir l'avis de plusieurs enseignant et normal que ceux ci se contre-dise mais cela à parfois considérablement ralenti notre travail. Une discutions entre eux et la recherche d'un commun accord , serait utile ici, vu la restriction de temps.
It is interesting to hear the opinion of several teachers and normal that they sometimes diverge -but this sometimes considerably slowed our work. Talking with each other and seeking common agreement, would be useful here, given the restrictions of time.

- Les avis très divergent des différents prof et à la fois intéressant mais aussi très déroutant surtout lors de la réalisation de la première séquence car nous n'avons pas encore l'habitude ni le recul sur ce qui est attendu et du coup on ne sais plus trop quoi penser
Opinions differ among different instructors which is both interesting but also very confusing especially when carrying out the first sequence because we have not yet used nor the experience on what is expected and we do not know what to think about this.

- Je trouve cette matière assez intéressante mais savoir exactement les métiers où elle débouche et que faire avec dans l'avenir, est ce réellement un métier d'avenir? Cela reste assez flou pour moi mais je reste optimiste dans l'ensemble.
I find this course fairly interesting but it is unclear what the future perspective are; is this really a job for the future? It remains unclear to me but I remain optimistic.

- La quantité de travail est raisonnable si elle est équitablement partagée.
The amount of work is reasonable if it is equitably shared.

- Il faudrait juste que les correcteurs se mettent d'accord à propos de certains éléments car parfois

l'un nous dit que c'est bon et l'autre nous dit le contraire et c'est un peu déroutant car quand on passe des heures à le faire et que le deuxième nous dise qu'il ne fallait pas faire ça ça nous met un coup dans le moral.
Instructors should agree about some elements because sometimes one tells us it's good and another says the opposite and it is a little confusing because when you spend hours on something and that the second instructor says that we should not do this, this is discouraging.

- Le gros défaut de l'Annotathon est que suivant le professeur qui corrige la note n'est vraiment pas la même. Il y a une inégalité au niveau de la rigueur de correction. De plus vos opinions divergent d'une correction à l'autre. Sinon cette matiére est intéressante et très plaisante. Merci à vous.
The major defect of the Annotathon is that the evaluator is not always the same. There is inequality in the accuracy of evaluation. And your opinions diverge from one to another. However this course is interesting and very pleasant. Thank you.

- La double correction est une excellente choses car les correcteurs ne verront pas les mêmes erreurs. Cependant, réappliquer toutes les consignes données par un enseignant et avoir une note très moyenne est très déroutant et fait sans doute plus de mal que de bien. Oui a deux opinions mais non à deux attentes différentes.
The double evaluation is a great things because the evaluators will not see the same mistakes. However, applying precisely all instructions given by a teacher and have a very average grade is very confusing and does probably more harm than good. Yes to two opinions but no to two different expectations.

- Pour les commentaires au niveau de deux enseignants, il faudrait que la personne qui nous donne le conseil nous corrige car quand les avis divergent on ne sais pas lequel prendre en compte et parfois on fait le mauvais choix === Mauvaise appréciation.
Concerning *comments made by two teachers, the person who gives us advice should also evaluate us because if opinions diverge we do not know which one to take into account and sometimes we made the wrong choice === Poor appreciation ...*

- Pour ma part ces TD m'ont permis de comprendre le cours et m'ont énormément fait progresser en bioinformatique. Cependant la quantité de travail est excessive (l'annotation des séquence prend beaucoup de temps surtout lorsque l'on en a jamais fait) et la note associée à cette UE n'est pas représentative du temps passé à annoter ces séquences). En ce qui me concerne aussi je trouve que le travail en binôme (enfin trinôme) n'est pas une bonne chose surtout quand on se retrouve seule à tout faire!.....
For my part these practicals allowed me to understand the course and make tremendous progress in bioinformatics. However, the amount of work is excessive (annotation of the sequence takes a long time especially if one has ever done this before) and the grade associated with the course is not representative of the time spent annotating these sequences). As for me, so I think the work in pairs (finally triad) is not a good thing, especially when you are left alone to do everything !.....

- La bio-informatique a été pour moi une grande découverte. Du coup ça été difficile et malgré le travail personnel que j'ai fourni pour cette année j'ai toujours autant de lacunes. Donc je ne pourrais pas poursuivre dans ce domaine pour un master mais ça peut toujours me servir.

The bio-informatics has been for me a great discovery. Therefore it was difficult and despite the personal work that I have provided this year I still have many gaps. So I could not continue in this field for a master but it can still prove useful.

- Il y avait trop peu d'enseignants par rapport au nombre d'élèves d'où une attente parfois longue avant d'avoir une opinion (donc possibilité d'avoir l'opinion de plusieurs enseignants difficile). Le fait que deux encadrants différents puissent dans certaines situations avoir des interprétations différentes joue sur la note. En effet l'un dit blanc et l'élève comprend et écrit blanc puis un autre encadrant arrive derrière, dit noir et l'élève est perdu. Je pense que le système de double correction est utile. Cependant il existe de trop grandes différences de notes entre les enseignants: il n'y a pas d'équité dans les notes. Les critiques de mes annotations ne m'ont pas faite progresser sur l'ensemble des TD. Certes l'annotation était meilleure après une seconde correction mais la première annotation fut meilleure que la seconde, elle-même meilleure que la troisième pour un contenu similaire.
  There were too few teachers compared with the number of students therefore we sometimes had to wait a long time before having an opinion (and thus getting the opinion of several teachers was difficult). The fact that two different supervisors may in certain situations have different interpretations influences the final grade. In fact, one says white, the student understands and writes white then another comes behind, says black and the student is lost. I think the double correction is useful. However there are too big differences between teachers marks: there is no equity in the marks. The criticism of my annotations did not help me to progress. While the annotation was better after a second correction but the first annotation was better than the second, itself better than the third for similar content.
