# Supplementary material for: Metagenome Annotation Using a Distributed Grid of Undergraduate Students
Source: PLoS Biol. 2008 Nov 25;6(11):e296. doi: 10.1371/journal.pbio.0060296 (PMC2586363; doi:10.1371/journal.pbio.0060296)
Supplement: Text S2 — (67 KB PDF). [file pbio.0060296.sd002.pdf]

Supplementary Text S2

List of students who took part in the 2005-2008 Annotathon campaigns:

ABRIC	AURÉLIE	BioCell 2005
AGUZZI	ANTHONY	BioCell 2005
AHMED	BOURHANE	BioCell 2005
AIMAR	SYLVAIN	BioCell 2005
ASCHTGEN	MARIE-STÉPHANIE	BioCell 2005
AVELLA	JEAN CHRISTOPHE	BioCell 2005
AVENTINY	JESSICA	BioCell 2005
BACHTARZI	MOHAMED	BioCell 2005
BAHIA	SADI ALI	BioCell 2005
BASTET	LAURENCE	BioCell 2005
BECK	VIRGINIE	BioCell 2005
BELLIARD	THIBAULT	BioCell 2005
BENASSILA	MAJDA	BioCell 2005
BENHAIM	ALEXANDRA	BioCell 2005
BENHAMOUD	ISAM	BioCell 2005
BERDIE	FLORENCE	BioCell 2005
BERENGUIER	DUNCAN	BioCell 2005
BIZEUL	AURELIEN	BioCell 2005
BOUDON	VIRGINIE	BioCell 2005
BOURRIER	EMILIE	BioCell 2005
BRACCINI	MURIEL	BioCell 2005
BRANDICOURT	MATHIEU	BioCell 2005
BROS	NATHALIE	BioCell 2005
CABANE	CHRISTELLE	BioCell 2005
CESSELIN	CÉLIA	BioCell 2005
CONIGLIARO	JOHANNA	BioCell 2005
COUMES	STÉPHANIE	BioCell 2005
COUVERT	CORALIE	BioCell 2005
DAHAN	DIANA	BioCell 2005
DARWICHE	NASRINE	BioCell 2005
DE VILLELE	SYLVAIN	BioCell 2005
DELLA CASA	SANDRINE	BioCell 2005
DELMASTRO	ALEXANDRA	BioCell 2005
DELPY	GAËLLE	BioCell 2005
DEMY	DORIS LOU	BioCell 2005
DESCOSTES	NICOLAS	BioCell 2005
DEYDIER	MARIE	BioCell 2005
DI MARINO	SANDRA	BioCell 2005
DJELLOUL	MEHDI	BioCell 2005
DRIDI	DOUNIAZED	BioCell 2005
EL CHAAR	TYAMA	BioCell 2005
EL KHOURY	LAYAL	BioCell 2005
FABRE	ELODIE	BioCell 2005
FAURE	AURÉLIE	BioCell 2005
FLORIS	MAINA	BioCell 2005
FRANCISCA	JEAN MARC	BioCell 2005
GANAY	CAROLINE	BioCell 2005
GARCIA	JULIEN	BioCell 2005
GASTON	ROBERT	BioCell 2005
GIDDE	NOÉMIE	BioCell 2005

GRANGE	MAGALI	BioCell 2005
GUENIOT MORIZOT	CAMILLE	BioCell 2005
GUIJARRO	LAURENT	BioCell 2005
GUYOT	VINCENT	BioCell 2005
HARDY	JULIEN	BioCell 2005
HAYER	JULIETTE	BioCell 2005
HIRCHAUD	FABIENNE	BioCell 2005
HUBERT	ANNE SOPHIE	BioCell 2005
HUBERT	LUCAS	BioCell 2005
ISPA	KARINE	BioCell 2005
JEANNIARD	ADRIEN	BioCell 2005
JOUVE	LORELINE	BioCell 2005
KHALFA	OUALID	BioCell 2005
KOZOULIA	ANAIS	BioCell 2005
LA PORTA	AURÉLIE	BioCell 2005
LALAYMYA	ISMAHEN	BioCell 2005
LANGLOIS	ANAÏS	BioCell 2005
LE GAY	GAËLLE	BioCell 2005
LE GUEUT	CLAIRE	BioCell 2005
LEPOIVRE	CYRILLE	BioCell 2005
LI	YUE	BioCell 2005
MAGALHAES	AUDREY	BioCell 2005
MAJGIER	CHARLOTTE	BioCell 2005
MANTILLERI	ANNABELLE	BioCell 2005
MARCHAND	YOHAN	BioCell 2005
MARKOVA	MARINA	BioCell 2005
MARTIROSSIAN	ANNA	BioCell 2005
MAURER	SONIA	BioCell 2005
MERBAH	MÉLANIE	BioCell 2005
MICOUD	VIRGINIE	BioCell 2005
MOLINA	THIERRY	BioCell 2005
MONTEIRO	NOÉMIE	BioCell 2005
MURATOT	SOPHIE	BioCell 2005
NICOT	MARION	BioCell 2005
OLIVE	BENOÎT	BioCell 2005
OUERFELLI	SABER	BioCell 2005
PASCAL	MAXIME	BioCell 2005
PETIT	EMILIE	BioCell 2005
PICCA	BENJAMIN	BioCell 2005
RAME	MARION	BioCell 2005
RAYNE	JULIEN	BioCell 2005
REVY	DELPHINE	BioCell 2005
RIBOULET	MURIEL	BioCell 2005
ROBERRINI NEVEU	GABRIEL	BioCell 2005
RODRIGUES	JULIE	BioCell 2005
RODRIGUES	OLIVIER	BioCell 2005
ROMMELAERE	SAMUEL	BioCell 2005
ROPERO	ALICE	BioCell 2005
ROUGEOT	JULIEN	BioCell 2005
ROUSSEL	LARA	BioCell 2005
ROVINI	AMANDINE	BioCell 2005
SCHWARTZ	MARIE-ELISE	BioCell 2005
SERAIN	NELLY	BioCell 2005
SOULAVIE	FABIEN	BioCell 2005

TALLON	MARINE	BioCell 2005
THOME	RÉMI	BioCell 2005
TRON	AMANDINE	BioCell 2005
VALLEJO	LIVIA	BioCell 2005
VANDENABEELE	SYLVIE	BioCell 2005
VANKEERBERGHEN	RÉMI	BioCell 2005
VERNEREY	JULIEN	BioCell 2005
VILMONT	VALÉRIE	BioCell 2005
YAO-N'DRE	MARINA	BioCell 2005
ACIEN	CAROLINE	BioCell 2006
ADALBERT	JULIE	BioCell 2006
AGNELLI	LAUREN	BioCell 2006
ALLARD	CLAIRE	BioCell 2006
ANGUILLE	FRÉDÉRIC	BioCell 2006
ARBEILLE	ELISE	BioCell 2006
ARFI	YONATHAN	BioCell 2006
ASSOUS	MAXIME	BioCell 2006
AZAIS	MARINE	BioCell 2006
BENARD	MATHILDE	BioCell 2006
BENHAMOUD	ISAM	BioCell 2006
BENIKHLEF	ZOUBIDA	BioCell 2006
BENNOUAR	PRÉNOM	BioCell 2006
BERTRAND	CHLOE	BioCell 2006
BEYLARD	EMMANUELLE	BioCell 2006
BLANC	GEOFFREY	BioCell 2006
BOEUF	DOMINIQUE	BioCell 2006
BOURDAT	MAGALIE	BioCell 2006
CAMPAGNA	CHRISTELLE	BioCell 2006
CASSAR	MARLENE	BioCell 2006
CATTENOZ	DIANE	BioCell 2006
CATTENOZ	JUDITH	BioCell 2006
CHABAUD	PIERRE	BioCell 2006
CHABERT	MARION	BioCell 2006
CLERAMBOURG	MAGALI	BioCell 2006
COMBEL	MAXIME	BioCell 2006
COUPAYE	LÉO	BioCell 2006
DENDOOVEN	ARNAUD	BioCell 2006
DI MARINO	SANDRA	BioCell 2006
DJEBAR	MOHAMED	BioCell 2006
DRIDI	DOUNIAZED	BioCell 2006
ENJALBERT	JULIE	BioCell 2006
ESTEVE	CLOTHILDE	BioCell 2006
ESTEVES VIEIRA	VERA	BioCell 2006
FARAH	HANAD	BioCell 2006
FAURE	CLAUDINE	BioCell 2006
FIESCHI	MAXIME	BioCell 2006
FONTAINE	MATHILDE	BioCell 2006
GAZON	HELENE	BioCell 2006
GIRAUD	HELENE	BioCell 2006
GRAVIER	CAMILLE	BioCell 2006
GUICHARD	CELINE	BioCell 2006
GUILLOUX	LOÏC	BioCell 2006
HONNORAT	CHARLOTTE	BioCell 2006
IBARES	AUDREY	BioCell 2006

INNOCENTI	CHARLENE	BioCell 2006
INNOCENZI	JULIEN	BioCell 2006
JANE	JONATHAN	BioCell 2006
JULIEN	RENAUD	BioCell 2006
KHALFA	OUALID	BioCell 2006
KIEFFER	VALÉRIE	BioCell 2006
LAFON	CLAIRE	BioCell 2006
LEONETTI	CHRISTELLE	BioCell 2006
LEYDIER	CHLOÉ	BioCell 2006
LEZEAU	SAMI	BioCell 2006
LISON	LESLIE	BioCell 2006
LOMBARD	JONATHAN	BioCell 2006
LORENZO	EDOUARD	BioCell 2006
MACHI	MARC	BioCell 2006
MALAVIEILLE	AURÉLIE	BioCell 2006
MARADJI	AMANDINE	BioCell 2006
MARILLY	SOPHIE	BioCell 2006
MARTINEZ	MAXIME	BioCell 2006
MASQUELIER	MARION	BioCell 2006
MEGY	MARION	BioCell 2006
MESQUIN	LAURELINE	BioCell 2006
MESURET	GUILLAUME	BioCell 2006
MORERE	JULIA	BioCell 2006
MY	LAETITIA	BioCell 2006
NOLFO	JULIE	BioCell 2006
NOUAR	ROQYA	BioCell 2006
OUEDRAOGO	DAVID	BioCell 2006
PAPANIAN	MARION	BioCell 2006
PARDOUX	ROMAIN	BioCell 2006
PETITJEAN	CELINE	BioCell 2006
PICARD	CYRIL	BioCell 2006
PRUDENT	ELSA	BioCell 2006
RAGHEB	RAMI	BioCell 2006
RAPIN	LAÉTITIA	BioCell 2006
RIBOULET	MURIEL	BioCell 2006
RICODEAU	NICOLAS	BioCell 2006
RODRIGUES-ELY	ALIX	BioCell 2006
ROLLAND	SARAH	BioCell 2006
ROUDAUT	YANN	BioCell 2006
SEKATCHEFF	VANESSA	BioCell 2006
SELLEM	JONATHAN	BioCell 2006
SIMONI	AURELIE	BioCell 2006
SPAGNOLO	JENNIFER	BioCell 2006
STRETTI	CHARLOTT	BioCell 2006
SULTAN	LIONEL	BioCell 2006
SUROY	MAXIME	BioCell 2006
VALENTE	MICHAEL	BioCell 2006
VERCHERAT	ERIC	BioCell 2006
VERNEAU	JONATHAN	BioCell 2006
VIARRE	REGIS	BioCell 2006
WARTEL	MORGANE	BioCell 2006
ZEPPONI	VANESSA	BioCell 2006
ZWOJSCZYKI	GAEL	BioCell 2006
ADDA BENATIA	AZIZ	BioCell 2007

ARGENTI	CLEMENT	BioCell 2007
ARMITANO	JOSHUA	BioCell 2007
BELAID	AMINE	BioCell 2007
BELHOCINE	MOHAMED	BioCell 2007
BENALLAL	FELLA	BioCell 2007
BENAROUCHE	ANAIS	BioCell 2007
BENSEMMANE	SHERIHANE	BioCell 2007
BOLIN	VIRGINIE	BioCell 2007
BONNET	EMILIE	BioCell 2007
BOURDAT	MAGALI	BioCell 2007
BOURGES	CHRISTOPHE	BioCell 2007
BOURHIS	AURELIE	BioCell 2007
BOUZIDI	SOFIA	BioCell 2007
BRUNET	YANNICK	BioCell 2007
BUI-VAM	AMANDINE	BioCell 2007
CADORET	FREDERIC	BioCell 2007
CAPASSO	CARINE	BioCell 2007
CAVOZZA	LAURE	BioCell 2007
CERESO	NICOLAS	BioCell 2007
CHAVE	DELPHINE	BioCell 2007
COMPAGNONE	MARION	BioCell 2007
CONNAN	CHLOE	BioCell 2007
CORRE	CEDRIC	BioCell 2007
COUMONT	CHARLOTTE	BioCell 2007
CREMADES	ADELINE	BioCell 2007
D'AMICO	NICOLAS	BioCell 2007
DAVID	MYLENE	BioCell 2007
DE FABRITUS	LAURIANE	BioCell 2007
DEL VECCHIO	ALEXANDRE	BioCell 2007
DEMOLLEINS	MARIE	BioCell 2007
DEPOORTER	FLORENT	BioCell 2007
DESMARAIS	DAMIEN	BioCell 2007
DISPA	CECILE	BioCell 2007
DJEBBAR	MOHAMED	BioCell 2007
ESTEVE	CHLOTILDE	BioCell 2007
FARADON	AURELIE	BioCell 2007
FERNANDEZ ORTEGA	EMILIE	BioCell 2007
FIEVET	ANOUCHKA	BioCell 2007
FRANCOIS	ANAIS	BioCell 2007
FRANCOU	ALEXANDRE	BioCell 2007
FUENTES	AURORE	BioCell 2007
GABISSON	JULIE	BioCell 2007
GAMBARELLI	GUILLAUME	BioCell 2007
GARCIA	FABIENNE	BioCell 2007
GARIBAL	MARC ANTOINE	BioCell 2007
GENSOLLEN	THOMAS	BioCell 2007
GHALLOUSSI	DORSAF	BioCell 2007
GHAZOUANE	RKIA	BioCell 2007
GONCALVES	AURÉLIE	BioCell 2007
GORVEL	LAURENT	BioCell 2007
GUEIT	NINA	BioCell 2007
GUEROULT-BELLONE	MARION	BioCell 2007
GUILLOT	CHARLENE	BioCell 2007
GYURIK	GWENAEL	BioCell 2007

HETHERRINGTON	PRIM	BioCell 2007
HOUARD	JESSICA	BioCell 2007
JACQ	VIOLAINE	BioCell 2007
JACQU0T	CAROLINE	BioCell 2007
JACQUET	LAUREEN	BioCell 2007
JAUSSAUD	EWALD	BioCell 2007
JUDD	KEVIN	BioCell 2007
KHALFA	OUALID	BioCell 2007
LAGET	AURELIE	BioCell 2007
LAMRAOUI	ADIL	BioCell 2007
LANA	GUILLAUME	BioCell 2007
LHERITIER	CHRISTOPHE	BioCell 2007
LLORENS	ANAIS	BioCell 2007
LORE	LAURIE	BioCell 2007
MALAVAL	MELANIE	BioCell 2007
MAZZELLA	JEAN MICHAEL	BioCell 2007
MDZOMBA	BAYA	BioCell 2007
MEILAC	THOMAS	BioCell 2007
MEMOUNI	RACHID	BioCell 2007
MEYER	ELODIE	BioCell 2007
MIGNOT	FLORIAN	BioCell 2007
MOHAMED	ZAITOUNI	BioCell 2007
NGKAMMAN	CHRISTELLE	BioCell 2007
OLIVARES	ORIANNE	BioCell 2007
OROSKO	JIMMY	BioCell 2007
PADUANO	VANESSA	BioCell 2007
PERET	ANGELIQUE	BioCell 2007
PIERONI	LUDIVINE	BioCell 2007
PISTILLI	BLANDINE	BioCell 2007
POGGIONOVO	CECILE	BioCell 2007
RE	STEPHANIE	BioCell 2007
ROCHE	BEATRICE	BioCell 2007
ROLLERO	STEPHANIE	BioCell 2007
ROSSI	ANNE LISE	BioCell 2007
ROUSSEL	JULIEN	BioCell 2007
ROUX	MARINE	BioCell 2007
ROUX	NICOLAS	BioCell 2007
SABER	AHMED	BioCell 2007
SAID SOILIHI	RIWADI	BioCell 2007
SAWKA	GREGOIRE	BioCell 2007
SCARPONE	MARIE	BioCell 2007
SCHWARTZ	JULIA	BioCell 2007
SEYRES	DENIS	BioCell 2007
SICHEZ	ADELE	BioCell 2007
SIMON	MARIE	BioCell 2007
SOLER	LUCIE	BioCell 2007
SUZAN	MAXIME	BioCell 2007
TONG	KIWEN	BioCell 2007
TRAORE	SY	BioCell 2007
TSALANLAL	AMEL	BioCell 2007
WEISS	EMELINE	BioCell 2007
AMIN ALI	OULFAT	Biochimie 2006
BARLETTA	MARION	Biochimie 2006
BERNARD	STÉPHANIE	Biochimie 2006

BLANCHARD	CÉCILE	Biochimie 2006
BOURHALFA	SOFIA	Biochimie 2006
CARBONNEL	LOUIS	Biochimie 2006
CHASTAN	FRÉDÉRIC	Biochimie 2006
CHOUATI	SAFAE	Biochimie 2006
CIACCAFAVA	ALEXANDRE	Biochimie 2006
CORFA	JULIE	Biochimie 2006
COTTA	JULIE	Biochimie 2006
D'AUBAS DE FERROU	CÉCILE	Biochimie 2006
DI MARIA	MARINA	Biochimie 2006
DRULA	ELODIE	Biochimie 2006
EL AAZZAOUI	SAMIRA	Biochimie 2006
GARCIA	MAXIME	Biochimie 2006
GAUSSEN	MARION	Biochimie 2006
GERMAIN	ELSA	Biochimie 2006
GOUMIDI	SOREYA	Biochimie 2006
GRAZIANI	SOPHIE	Biochimie 2006
GUEGUEN	MARION	Biochimie 2006
HED NOUIRE	BOINA	Biochimie 2006
LABAT	CHRISTINE	Biochimie 2006
LAPORTE	MARIE-ANGÉLIQUE	Biochimie 2006
LE HIR	JÉRÔME	Biochimie 2006
LEFLOCH	DOROTHÉE	Biochimie 2006
LI	TENGFEI	Biochimie 2006
LORENDEAU	DOROTHÉE	Biochimie 2006
LOUAI	SOUAD	Biochimie 2006
MAOULANA	SAID YOUSSOUF	Biochimie 2006
MARK	VANESSA	Biochimie 2006
MASSE	JULIE	Biochimie 2006
MBODJ	ABIBATOU	Biochimie 2006
MERABET	NABILA	Biochimie 2006
MILIOTO	CÉDRIC	Biochimie 2006
NDIAYE	CHEIKH TIDIANE	Biochimie 2006
NDIAYE	EMMANUEL	Biochimie 2006
NICOLAS	VIRGINIE	Biochimie 2006
ORDOACER	FLAVIE	Biochimie 2006
PHAM	NGUYEN BAO KHANH	Biochimie 2006
PLANTEVIN	FLORIANE	Biochimie 2006
POULIN	PASCALINE	Biochimie 2006
PREIRA	PASCAL	Biochimie 2006
ROBERT	LUDIVINE	Biochimie 2006
SCHWALL	MÉLISSA	Biochimie 2006
SETRICK	GUILLAUME	Biochimie 2006
SEVAJOL	MARION	Biochimie 2006
SUZANON	DIDIER	Biochimie 2006
SZYTTENHOLM	ALEXANDRA	Biochimie 2006
TA	THI ANH TU	Biochimie 2006
TOMAS	JULIE	Biochimie 2006
TRAORE	SY SANBLE WILFRIED	Biochimie 2006
VANDEVELDE	MANUEL	Biochimie 2006
VILLARD	MÉLANIE	Biochimie 2006
YANG	YUN XUAN	Biochimie 2006
ZAHARATOUN	CORINNE	Biochimie 2006
ZHANG	CHI	Biochimie 2006

ABELLA	MARIE-PIERRE	Biochimie 2007
ABI SAAD	NADIM	Biochimie 2007
AMBRAISSE	ELODIE	Biochimie 2007
ANDRIAMANJAY	ANGISITRAKA HAMY	Biochimie 2007
ANTONI	HADRIEN	Biochimie 2007
AUSSIGNARGUES	CLÉMENT	Biochimie 2007
BALIQUE	FANNY	Biochimie 2007
BAYLOT	VIRGINIE	Biochimie 2007
BENESIN	AURORE	Biochimie 2007
BENGHERBIA	MONIA	Biochimie 2007
BLANCHARD	CÉCILE	Biochimie 2007
BONGIORNO	MAXIME	Biochimie 2007
CANO	MELISSA	Biochimie 2007
CECCALDI	PIERRE	Biochimie 2007
CIACCAFAVA	ALEXANDRE	Biochimie 2007
DADI	GUILA	Biochimie 2007
DAGUERRE	YOHANN	Biochimie 2007
DEWASCH	RÉMI	Biochimie 2007
DIOUET	RACHEL	Biochimie 2007
ELAID	SARAH	Biochimie 2007
GIRAUD	EVA	Biochimie 2007
GOHEBEL	SYLVIA	Biochimie 2007
GOTTHARD	GUILLAUME	Biochimie 2007
GUIRAL	CLÉMENCE	Biochimie 2007
HAMIDOU BOINA	NOURA	Biochimie 2007
HARICHANE	KARIMA	Biochimie 2007
INNOCENT	THIBAULT	Biochimie 2007
KHALLADI	HAYATE	Biochimie 2007
LEONETTI	VANESSA	Biochimie 2007
LIBBRA	JULIEN	Biochimie 2007
LUBIN	LOLITA	Biochimie 2007
MADI	DARKAWI	Biochimie 2007
MANEVILLE	STÉPHANIE	Biochimie 2007
MARK	VANESSA	Biochimie 2007
MASSON	VINCENT	Biochimie 2007
MEDINA	JONATHAN	Biochimie 2007
MEGY	FLORIE	Biochimie 2007
MIMOUNI	JOHANNA	Biochimie 2007
MOHAMED	ZOUWINA	Biochimie 2007
NISSIOITI	TAMINE	Biochimie 2007
OROMATO	AURÉLIE	Biochimie 2007
PETER VALENCE	FRÉDÉRIQUE	Biochimie 2007
PILOURDAULT	SOPHIE	Biochimie 2007
ROURE	CLÉMENCE	Biochimie 2007
SERGEANT	AURÉLIE	Biochimie 2007
STAINER	KARINE	Biochimie 2007
TADJDET	MOUNA	Biochimie 2007
TERRASSE	RACHEL	Biochimie 2007
TRAORE	SY SANBLE WILFRIED	Biochimie 2007
WANG	LE	Biochimie 2007
XU	GUANG JUN	Biochimie 2007
ZAHARATOUN	CORINNE	Biochimie 2007
ZHAO	QIUSHI	Biochimie 2007
ARNAUD	MARINE	Biochimie 2008

AUGE	CAMILLE	Biochimie 2008
BAHBAH	NADJAT	Biochimie 2008
BAKARI	EN-ICHAT BINTI	Biochimie 2008
BANAOUAS	AHLEM	Biochimie 2008
BEN MAHJOUBA	KHANSA	Biochimie 2008
BENKADDOUR	INTISSAR	Biochimie 2008
BLANC	JEAN MICHEL	Biochimie 2008
BONNEAU	ALINE	Biochimie 2008
BONZI	JEREMY	Biochimie 2008
BOUTALEB	MAYA	Biochimie 2008
CARLETTINI	HELENE	Biochimie 2008
CASTELLI	DAMIEN	Biochimie 2008
CHADULI	DELPHINE	Biochimie 2008
CHEILAN	FLORIE	Biochimie 2008
COMBES	MAUD	Biochimie 2008
CORRIGER	LISE	Biochimie 2008
DALI	TOUFIK	Biochimie 2008
DE PABLOS	CÉCILE	Biochimie 2008
DERREZ	ESTELLE	Biochimie 2008
DJEBALI	HANA	Biochimie 2008
EZ-ZAHER	NORA	Biochimie 2008
FERNANDEZ	MANUEL	Biochimie 2008
FRAISSE	MARIE EVE	Biochimie 2008
FRANCOIS	BENEDICTE	Biochimie 2008
FRITZ	CHARLES	Biochimie 2008
GARGUILO	MARIE CAROLINE	Biochimie 2008
GIL	JEAN-CHARLES	Biochimie 2008
GOUIRAN	CAROLINE	Biochimie 2008
HAMIDOU BOINA	NOURA	Biochimie 2008
HERPIN	TIPHAINE	Biochimie 2008
IMBERT	CORALIE	Biochimie 2008
KHOUGAZIAN	IVAN	Biochimie 2008
KOGUT-KUBIAK	TATIANA	Biochimie 2008
KOUDOUSSI	SIHAME	Biochimie 2008
KOURJIAN	GEORGIO	Biochimie 2008
LAFFONT	BENOIT	Biochimie 2008
LI	TINGTING	Biochimie 2008
LONGHITANO	JONATHAN	Biochimie 2008
LUCHAIRE	MARION	Biochimie 2008
MALAOUI	IMANE	Biochimie 2008
MALAOUI	SAFA	Biochimie 2008
NDIAYE	MOUSTAPHA	Biochimie 2008
NSIGUE MEILO	SANDRINE VANESSA	Biochimie 2008
PUSCHIASIS	AUDREY	Biochimie 2008
RIBOULET	ELODIE	Biochimie 2008
TACHOUAFT	MELISSA	Biochimie 2008
TRIKI	AMIRA	Biochimie 2008
ZUCCHELI	VANESSA	Biochimie 2008
BOSSARD	MAUD	ESIL 2006
BOUBIS	BENJAMIN	ESIL 2006
BUTTIGIEG	DOROTHÉE	ESIL 2006
CAMEAU	EMMANUELLE	ESIL 2006
CHAULIAC	DIANE	ESIL 2006
DANIEL	GUILLAUME	ESIL 2006

DESOIZE	ISABELLE	ESIL 2006
DUMOULIN	CHLOÉ	ESIL 2006
DUVAL	ANAÏS	ESIL 2006
ERTEL	GISELA	ESIL 2006
FAUCILLON	CÉCILE	ESIL 2006
GLUAIS	LAURE	ESIL 2006
HUGHES	JENNIFER	ESIL 2006
HUGHES	NICOLA	ESIL 2006
INVITÉ(E)	1	ESIL 2006
INVITÉ(E)	2	ESIL 2006
JEANNOT	ALINE	ESIL 2006
LANDRY	JONATHAN	ESIL 2006
LEFEBVRE	CORALIE	ESIL 2006
LETOQUART	JULIETTE	ESIL 2006
LICARI	CHRISTOPHER	ESIL 2006
LUGARI	ADRIEN	ESIL 2006
MARCEAU	NICOLAS	ESIL 2006
MARMIN	LAURE	ESIL 2006
MARTINE	ALEXANDRA	ESIL 2006
MASSE	ANTOINE	ESIL 2006
MERCIER	SARAH	ESIL 2006
MORETTI	NICOLAS	ESIL 2006
OUBELAID	RACHID	ESIL 2006
PETITJEAN	CHRISTOPHE	ESIL 2006
PIZZO	MAGALI	ESIL 2006
RAVISE	ALINE	ESIL 2006
REUSSER	DIANE	ESIL 2006
ROCHA	AURÉLIE	ESIL 2006
ROZOT	VIRGINIE	ESIL 2006
SEASSAU	AURÉLIE	ESIL 2006
SOUMILLON	MAGALI	ESIL 2006
THAMBIRAJAH	SATHIESAN	ESIL 2006
TRAVERSE	MARION	ESIL 2006
UZAN	EVA	ESIL 2006
VILLAMAUX	ANAÏS	ESIL 2006
